# Design of a Multiband Global Navigation Satellite System Radio Frequency Interference Monitoring Front-End with Synchronized Secondary Sensors

**DOI:** 10.3390/s18082594

**Published:** 2018-08-08

**Authors:** Aiden Morrison, Nadezda Sokolova, James Curran

**Affiliations:** 1 SINTEF, 7034 Trondheim, Norway; Nadia.Sokolova@sintef.no; 2 ESTEC (The European Space Research and Technology Centre), 2201 AZ Noordwijk, The Netherlands; jamestcurran@ieee.org

**Keywords:** GNSS signal interference, jamming, spoofing, inertial measuring units

## Abstract

This paper investigates the challenges of developing a multi-frequency radio frequency interference (RFI) monitoring and characterization system that is optimized for ease of deployment and operation as well as low per unit cost. To achieve this, we explore the design and development of a multiband global navigation satellite system (GNSS) front-end which is intrinsically capable of synchronizing side channel information from non-RF sensors, such as inertial measurement units and integrated power meters, to allow the simultaneous production of substantial amounts of sampled spectrum while also allowing low-cost, real-time monitoring and logging of detected RFI events. While the inertial measurement unit and barometer are not used in the RFI investigation discussed, the design features that provide for their precise synchronization with the RF sample stream are presented as design elements worth consideration. The designed system, referred to as Four Independent Tuners with Data-packing (FITWD), was utilized in a data collection campaign over multiple European and Scandinavian countries in support of the determination of the relative occurrence rates of L1/E1 and L5/E5a interference events and intensities where it proved itself a successful alternative to larger and more expensive commercial solutions. The dual conclusions reached were that it was possible to develop a compact low-cost, multi-channel radio frequency (RF) front-end that implicitly supported external data source synchronization, and that such monitoring systems or similar capabilities integrated within receivers are likely to be needed in the future due to the increasing occurrence rates of GNSS RFI events.

## 1. Introduction

This study has focused on the twin objectives of sensor system development in pursuit of producing a GNSS front-end or bit grabber that solves the problems of accurately synchronizing the collected data at the sample level with other navigation sensors, while also providing for a cost-effective means of detecting and characterizing RFI events in the GNSS spectrum.

The existing strike3 undertaking (www.gnss-strike3.eu) is a European Horizon 2020 project directed at the standardization of GNSS threat reporting and receiver testing, running from 2016 through 2019, that has some overlap with the goals of this study, but has a stronger focus on the creation of a centralized database of searchable metrics of the detected interference events [1].

While the production of this very high-quality data in the sense of it utilizing multi-bit quantization and automated characterization and finger printing worked as expected, one area of weakness in the STRIKE3 effort is that its minimum criteria for interference monitoring covers only a small subsection of the L1 band, not even ensuring that the wider Galileo binary offset carrier (BOC) components are covered, nor the public regulated service (PRS) on E1. Per the STRIKE3 reporting standards requirement [2] ID “STRIKE-MON-FUN-001” ‘The STRIKE3 monitoring systems shall detect interference in at least GPS L1 band’. Since many of the partners in the program are deploying Spirent GSS100D interference detectors with bandwidths of only 16 MHz around the Global Positioning System (GPS) L1 and 9 MHz around the GLObal NAvigation Satellite System (GLONASS) L1, the vast majority of GNSS signals are unmonitored by many STRIKE3 installations, despite the monitor being a large and expensive piece of rackmount equipment. On this hardware, jam and spoofing alerts are also apparently not supported per SPIRENT datasheets.

To achieve detection of RFI events, some authors advocate the use of a software defined radio (SDR) approach to not only the analysis of captured events, but also to their detection including Ying et al. [3], which has the advantages of producing metrics, such as the position domain disturbance magnitude during detected events, but such approaches have related drawbacks. The first obvious drawback is that SDR processing must trade-off the processing power and complexity of the collection system versus the number of signals and amount of spectrum to be monitored. The second apparent drawback is that the SDR-based system will require continuous operation and therefore power use of the processing system to evaluate the collected spectrum. The approach used by the FITWD diverges from this by pursing a tactic similar to the measurement of the automatic gain control (AGC) as investigated by References [4,5] and others, but instead integrates a wideband absolute RF power meter to provide coverage of both in-band and adjacent band interference. In principle this approach allows for the detection of abnormal RF emissions within the bands explicitly captured and digitized by the mixing, filtering, and digitization stages, but also those that occur in the adjacent bands and may be worthy of investigation. Simultaneously the approach used by the FITWD allows for arbitrarily large signal capture bandwidth without an increase in the required real-time processing capability as is needed by SDR based approaches.

While many other front-end systems exist, each has one or more drawbacks in the context of this application. Systems sold by National Instruments are capable of producing high bit-depth samples at sampling rates over 20 MHz, but their PXI [6] chassis, controllers, mixers, and digitizers are one or more orders of magnitude separated in each of size, weight, power consumption, and cost (SWAP+C). Some systems exist that leverage multichannel integrated tuners, such as the Amungo NUT4NT [7], though due to the limitations of the NT1065 [8], their gains in terms of the integration of four channels and two-bit digitization in to a single integrated circuit (IC) are offset by limited frequency flexibility (due to having only two independent phase locked loops (PLLs) for four channels) between the channels compared to the FITWD, which has fully independent PLLs per channel, in addition to not supporting integrated side-channel sensors. The somewhat inflexible NUT4NT and the extremely capable SdrNav40 [9] provide for phased array antenna operation at the cost of requiring external cabling and splitters for single antenna applications. In addition to supporting four completely flexible channels on a single board like the FITWD, the SdrNav40 is capable of synchronous operation of multiple boards to form larger arrays, and the disciplining of externally connected oscillators via a special interface. The SdrNav40 has several advantages over the FITWD in terms of the capabilities of the integrated field programmable gate array (FPGA) to perform re-sampling and filtering operations, support for external high stability oscillators, and capability to synchronize the RF sample streams with other SdrNav40s to form larger antenna arrays. However, it does require more power and space, is believed to have a bill of materials at least triple that of the FITWD due to the use of traditional analog to digital converters for each channel, has a much higher grade of FPGA, and is unfortunately no longer commercially available. High performance front-end systems from iFen [10] provide four physical channels of synchronized multi-bit samples, a software receiver solution, and the ability to synchronize side-channel sensors similar to the FITWD, though at a higher SWAP+C and seemingly without the frequency hopping support of the FITWD design [11]. This paper will focus on the hardware design of the monitoring front-end, the firmware and data structure optimizations used to achieve high throughput at low unit cost, the methods used for RFI event monitoring, and will conclude with examples of a detected event within a restricted research campus. While the design of the FITWD includes several elements that are not directly used in the GNSS RFI monitoring configuration, such as the integrated IMU plus magnetometer and barometer, the integrated tactical grade micro electro mechanical systems (MEMS) IMU interface, and the phase-coherent frequency hopping capabilities, the design techniques that allow these features are also essential for the RFI monitoring approach used here and will therefore be covered in detail. Briefly, it is worth commenting the potential applications of these sensors with specific emphasis on interference monitoring.

Naturally, in the context of a static installation, the addition of motion sensors is of no benefit; however, in mobile monitoring applications, the addition of motion sensors can provide a variety of benefits. When attempting to document the occurrences of interference and their locations, some positioning information is required, but can be denied to a GNSS-only user in the case that the interference causes a complete GNSS loss. The addition of inertial sensors can help to bridge brief GNSS outages and can preserve position accuracy in cases where a reduced number of satellites are tracked. When attempting to characterize interference, it can be of interest to discern between interference signal variations caused by modulation versus those caused by relative motion of the sources and the monitoring station. Inertial sensors can provide a very simple, robust, and always available means of discerning between static and moving (i.e., parked or driving monitoring) cases. A third, more advanced application of inertial sensors is the use of synthetic aperture antenna arrays for interference localization. A single antenna element moving along a known trajectory can be used to form a synthetic antenna array, which can provide angle of arrival information. In the case of a vehicular monitoring station, the addition of well-synchronized inertial measurements can be used to achieve reasonably high horizontal angle-of-arrival information, which in turn can be used for interference localization. Although, at present, these particular features are not yet utilized, the design choices that allow their inclusion make their consideration worthwhile.

## 2. Materials and Methods

Here we will describe the component elements of the overall FITWD design and how they relate to the goal of producing a low cost yet high capability RFI monitoring system in both function and conceptually. Since the RFI monitoring application is a layer of software running in support of multi-purpose hardware, we will start with the underlying hardware. The overall capabilities and adjustable parameters of the FTIWD are listed in Table 1, along with bolded values that indicate the specific settings used for the data collection campaign and results discussed.

### 2.1. Hardware Design

**Figure 1:**
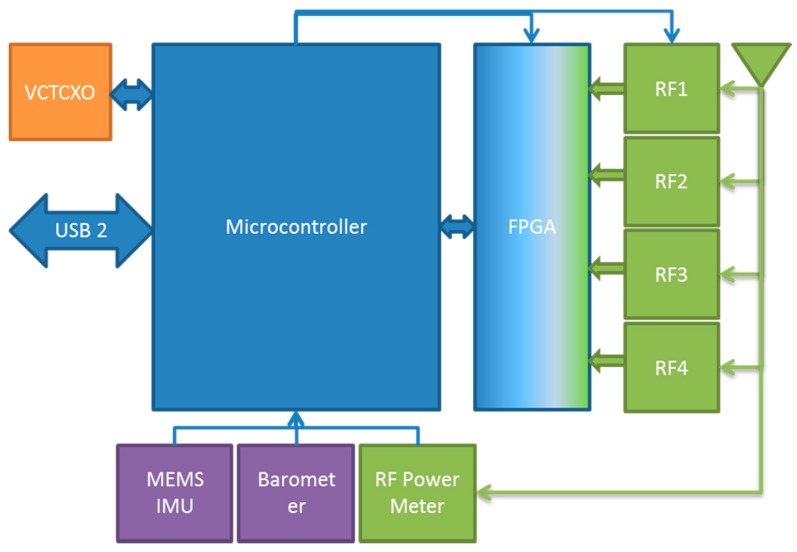
FITWD system block diagram per Reference [11].

The FITWD hardware comprises several discrete components as depicted in Figure 1, where analog or RF elements are color-coded green, digital components in the signal path are color-coded dark blue, peripheral digital sensors are purple, and the clock subsystem is color-coded orange.

#### 2.1.1. RF Signal Handling

The most critical design element of a GNSS front-end is the first stage of RF signal handling. This is a consequence of the extremely low power level of the expected received signals, and while the use of an active antenna can provide tens of decibels of gain, the received power level is still expected to be in the vicinity of −100 dBm, or 10 to 50 dB weaker than a typical WiFi signal. For this reason, the antenna connector attaches to a 50 Ω coplanar waveguide to the input stage of a Macom Maal-011078 [12] wideband low noise amplifier (LNA). While a direct current (DC) blocking capacitor is installed serially with this trace to protect the input of the LNA from the 5 V DC injected in to the antenna feed-line to power the antenna LNA, each of these three functions is carried out within a distance of 5 mm from the tip of the subminiature A (SMA) connector to the RF input pin of the LNA to minimize losses.

The Macom LNA was selected to provide both an operational gain band larger than required for GNSS applications, but also to support a relatively high gain of approximately 28 dB and a third order intercept point well beyond the nominal power level at the output of an active antenna of +30 dBm. Given that the expected input power level from an active antenna is in the vicinity of −100 dBm, the analog gain stage can tolerate nearly 13 orders of magnitude of RFI power before analog distortion becomes dominant, though it may suffer physical damage slightly before this point. To minimize the impact of unintentionally amplifying strong out-of-band signals by 28 dB in the LNA, the output of the LNA was connected to a Walsin RBBPF3225180C67B1U [13] ceramic band-pass filter that provides an insertion loss of 2 dB over the 1125–1675 MHz range, and over 40 dB of attenuation below 1 GHz and above 2 GHz. While the antenna and its intrinsic filtering or added internal filters must provide the first stage of frequency selectivity, the addition of the post-gain filter helped ensure that out-of-band signals were at worst no stronger relative to the signals of interest than they were at the active antenna output, which can otherwise happen due to the LNA providing slightly higher gain outside of the normal GNSS frequency ranges than it does within.

Routing and division of the filtered RF signal was carried out via 50 Ω impedance-matched coplanar waveguides tuned for the specific characteristics of the printed circuit board fabrication process used, and Anaren PD0922J5050S2HF [14] 50 Ω 1:2 power dividers. The filtered RF signal was symmetrically split twice to produce four equal signal taps. Three of these taps were impedance-matched to the inputs of the RF mixers, while the fourth was divided once more to provide a feed to the final RF mixer as well as the direct RF power measurement IC.

**Figure 2:**
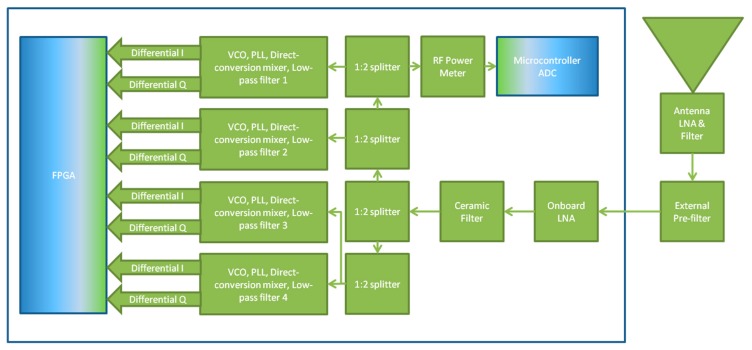
FITWD RF subsystem diagram. Elements in blue indicate sampling and digitization.

An expanded view of the RF signal handling components of the FITWD along with the externally connected antenna and filtering used in the GNSS RFI monitoring application are shown in Figure 2. Within this figure, the voltage-controlled oscillator (VCO), phase locked loop (PLL) direct conversion mixer, and low-pass filter blocks contain the 50:75 Ω conversion needed between themselves and the 1:2 splitters.

#### 2.1.2. RF Signal Measurement and Conversion

Since each of the signal division stages can be expected to have an insertion loss of 0.8 dB, plus the 3 dB loss from the equal two way power division, and the initial 2 dB loss from the ceramic filter the RF power measurement, the IC experiences a total loss of 13.4 dB plus residual losses in the printed circuit board (PCB) coplanar waveguides. While the equivalent power level for a single GNSS satellite signal would at this point be approximately −85 dBm, the total in band power would be dominated by the noise figure of the system antenna, and in practice would measure approximately −60 dBm in this implementation with a tri-band antenna such as the Novatel 850 [15]. To allow power measurement over a wide dynamic range that includes this minimum, the Linear Technology LT5538 RF power detector [16] was selected as the measurement device, providing a −72 dBm minimum and 75 dB linear dynamic range. The output of the RF detector was low pass filtered before being digitized by the FITWD system microcontroller and packaged with the other system peripheral sensors as described later.

Signal conversion from RF to IF was accomplished through the use of Maxim MAX2120 [17] single stage down converters, which were selected due to their high flexibility and performance, which comes from their integration of a variable gain amplifier (VGA), a digitally programmable integer-N PLL driven mixer, and programmable sixth order filters. Since the intended market of the MAX2120 is satellite television, the devices make use of a 75 Ω input impedance and have a relatively high noise floor of up to 8 dB depending on their VGA voltage/gain setting, but these factors were easily worked around through an impedance-matching resistor pair at the input and the inclusion of the first stage LNA previously discussed, which makes the noise level contribution of this amplifier negligible. The output of the mixers was a pair of differential in-phase (I) and quadrature-phase (Q) signals with a differential voltage level of up to 2 volts peak-to-peak and a frequency band selectable in 1 MHz increments via the internal PLL and mixer.

#### 2.1.3. Sampling, Quantization, and Data Packing

To minimize the physical footprint and cost of the FITWD, while also minimizing the amount of downstream data produced during event detection and logging, the decision was made to restrict the design to single-bit quantization. As a result of this decision, it was not necessary to employ a discrete analog-to-digital converter for each I/Q branch of each channel, and it was instead possible to use the existing differential I/O cells of the system FPGA as single bit quantization stages. In addition to this quantization task, the Xilinx XC3S50A FPGA [18] was also used to implement a number of configurable features such as the selection of the sampling clock source from a fixed 24 MHz reference directly from the system voltage controlled temperature compensated crystal oscillator (VCTCXO), or a divisible 216 MHz source, as well as selecting 2- or 4-channel operation, full rate or halved sampling rates, and packing of data from the 8 bit source width (four channels of I/Q at 1 bit quantization) to 16 bit words for low rate handling by the system controller.

#### 2.1.4. Secondary Sensor Synchronization

Synchronizing the secondary sensors with the RF samples was accomplished through a series of design choices, and the exploitation of available peripherals within the Microchip PIC32MZ system controller [19]. The first and most important design choice that facilitated synchronization was that all activities within the system controller, FPGA and RF portions, were synchronously driven by a common clock source. The 24 MHz VCTCXO signal was buffered and routed to the RF section where it was used as the reference clock for the RF mixer PLLs, as well as to the digital section where it was used as the reference for the microcontroller PLL that multiplies it by a factor of nine to produce the 216 MHz system clock. Depending on the selected sampling frequency, either this derived system clock, the base 24 MHz system clock, or either of these divided by two were used to drive the signal sampling process, ensuring that a fixed ratio of reference clock cycles to sampling clock cycles existed. Simultaneously the 24 MHz reference clock was used to drive a 32-bit system timer which is enabled within one sample period of the start of signal sampling.

Since secondary sensors are often driven by their own internal clocks that are beyond control of the designer, and asynchronous with other parts of the system, it is in these cases desirable to apply a time stamp on the common system time scale to the epochs of data availability from the secondary sensors. This imposes the requirement that the selected secondary sensors should produce a signal, typically a rising or falling edge on a control line, to indicate to the system controller the epoch when data is valid, or ready for readout. In the former case the device produces a reference marker that indicates the epoch of the data exactly, while in the latter case it only indicates the slightly delayed epoch at which the data can be retrieved. In either case the FITWD system controller utilized a peripheral known as “input capture” whereby edge events on a controller pin triggered the logging of the value of the 32 bit system timer at the arrival of that edge. Since the timer was synchronous with the known ordinal RF sample number or known multiple thereof present at the start of every packet of data, and the logged epoch of the secondary sensor events was provided with a precision of one RF sample (or better in the case of lower sampling rates), the epoch of data or data availability from the secondary sensors is therefore known within one RF sample.

An intended modification to the next version of the FITWD will be the inclusion of a clock-gating function in the system FPGA such that the system controller can effectively handshake the beginning of sampling without the current residual unknown latency of 0–4 ticks of the 216 MHz system clock due to the pipeline of the controller. With that modification made, the synchronization of the secondary sensors would be known to be within 0–1 ticks of the 216 MHz system clock.

#### 2.1.5. Data Formatting and Sensor Data Side-Channel

**Figure 3:**
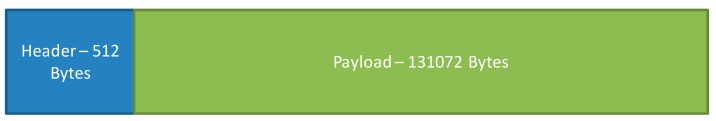
FITWD data packet diagram. Secondary sensor data was carried in the header.

The system controller, a Microchip PIC32MZ, received 16-bit words of data from the system FPGA and passed them in to buffers via direct memory access (DMA) control, with a slight twist. The region of memory mapped for use for RF sample packing was divided into blocks of 131,584 bytes, of which only 131,072 bytes were configured for the reception of samples streaming from the system FPGA. The remaining 512 bytes was configured as a header to form a packetized data format as per Figure 3.

While the data contained in the payload section was simply repeated 16-bit words of RF samples, the data in the header section was adaptable depending on the operating state and configuration of the front-end, and included several potentially novel features in the context of GNSS-focused front-ends. The header was initially flagged by a series of synchronization bytes to indicate the start of a header plus payload frame. Next, state information was included, such as the header length, the payload length, and the configuration data for each active RF channel, and the system overall making the logged data center frequency and configuration implicit to anyone analyzing the file. This feature was added to alleviate the difficulty of data control involving configurable RF sources in terms of archiving stream metadata alongside the RF samples or risking loss of data sets, or using large amounts of time to guess and check what center frequency and sampling rate might have been in use. This was especially important as the FITWD could be configured to support center frequencies with 1 MHz step sizes, filter bandwidths with approximately 290 kHz sizes, a multitude of sampling rates, and a variety of oscillator pulling, antenna power, and other options.

The next potentially novel feature was a synchronization word that indicated the sample number of the first RF sample in the payload within a free-running 32-bit word, which served as the point of synchronization between the RF sample stream and all secondary sensors running on the system that are time-stamped using this same free-running counter. While this 32-bit word will overflow roughly every 3 min when using a 24 MHz sampling rate, it provided sufficient range to synchronize the RF samples with the secondary sensors at the resolution of a single RF sample.

The secondary sensors were then embedded with headers, payloads, and footers of their own within the remaining approximately 480 bytes of the header structure using a cascaded priority configuration whereby messages were inserted in order of message type priority as long as space in the current header remained. For example, since the primary application is of RFI event detection, the RF power meter’s digitized output was packaged and inserted first using a header that indicated its presence and a footer for cyclic redundancy check (CRC) purposes. Next, barometric pressure and temperature messages were inserted, followed by an inertial measurement unit (IMU) and its integrated magnetometer messages until all messages were packaged or space within the header had been exhausted.

**Figure 4:**
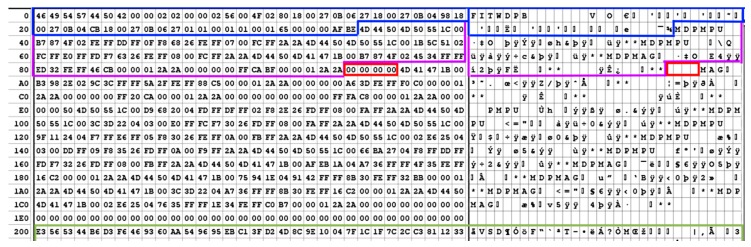
Hexadecimal (**left**) and ASCII (**right**) representations of an example FITWD header and start of RF payload.

To optimize the embedded processors workload, an “end of data” termination string consisting of two or four repeated zeros was used, such that the embedded controller did not have to “blank” the entire 512-byte header after every packet was sent, and instead only had to zero a few extra bytes during the data insertion step. An example of the FITWD data format and packing strategy is shown in Figure 4, where an entire header is presented up to byte 512 (200 hex), with the following packed RF samples demarcated with a green line. Within the header, the preamble and system configuration data for each of the RF channels, and the sampling rate, filter settings, antenna power configuration, etc. are encapsulated in dark blue. Within the purple bordered section are two inertial messages with headers “MDPMPU” and one magnetometer payload with header “MDPMAG”. Following the valid messages packed within the header is the repeated zeros marker bounded in red indicating where valid data within the current header ends. Since the repeating zero pattern was only looked for in lieu of a valid message header, such as “MDPMPU”, the presence of repeated zeros elsewhere in the message did not lead to framing problems. Bytes not encapsulated in any of the colored boundaries represent space that was not used in this particular packet, but the presence of fragments of previous messages indicated both where the optimization strategy had saved time, if not memory space, for the system controller, while the array of zeros indicated a sort of “high water mark” for the maximum variable packing strategy use level seen in this example.

This variable packaging strategy was necessitated by the variable header rate, which is in turn a consequence of the variable sampling rate. At 24 MHz sampling of four channel data a new packet was generated at a rate of approximately 92 Hz providing a side-channel bandwidth of slightly less than 43 kilobytes. At a 2 MHz sampling rate of two channel data, the available bandwidth for peripheral sensors would fall to just over 3.5 kilobytes, which likely resulted in the loss of events from the lowest priority data stream, in this case the magnetometers.

**Figure 5:**
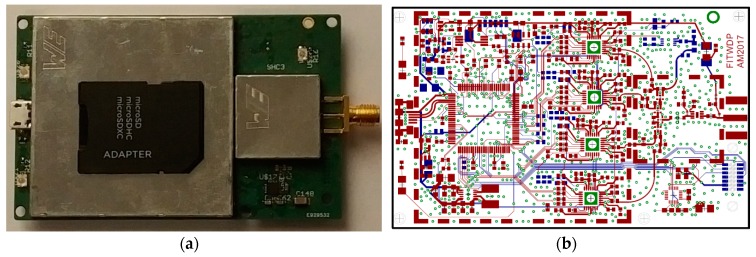
(**a**) FITWD with secure digital card for scale; (**b**) FITWD PCB showing shielded details of top/bottom layer.

Through such judicious optimizations, the four channel highly configurable front-end with multiple synchronized secondary data sources had fit into a footprint of only 8.5 by 5.4 cm, or that of a common bank card, as shown in Figure 5.

The motivation for the inclusion of upper and lower layer shielding over much of the FITWD, per Figure 5a, is that on such a small form factor, it proved difficult to avoid unintentional L-band emissions from the digital portions of the system. For this reason, virtually all of the active circuitry on the FITWD was enclosed within two large enclosures on the top and bottom of the board, with the first stage RF gain and splitting stages enclosed in a smaller enclosure seen in Figure 5a.

#### 2.1.6. Real-Time Monitoring

Due to the inclusion of synchronized RF power level measurements directly within the FITWD header structure, the system software responsible for monitoring only needed to parse a small amount of data from every packet to determine if an RF power level event has occurred, greatly reducing real-time processing power requirements compared to systems that use SDR for event detection directly. The FITWD was designed to support monitoring of any combination of L-band frequencies through the selection of attached antenna and pre-filter combinations. In the currently used configuration including the onboard and external pre-filters described in Table 1 that exclude all signal bands except L1 and L5 plus the roll off region of the filter adjacent to these bands, the system monitored only the parts of the spectrum where increased power emissions were a potential concern to GNSS L1 and or L5 operation. On power-up and reception of a valid configuration string, the FITWD began streaming collected spectrum samples as well as packed sensor data to the monitoring software running on the attached system (in this study a simple Raspberry Pi3). The monitoring software on the Pi3 began a local RF power environment characterization using the packed RF power meter sensor data from the FITWD, which takes into account the presence of fixed in-band or adjacent-band emitters, such as radars or direction measuring equipment (DME), in its determination of the local typical noise level. While this stage ran on the Pi3 for only 90 s, the software then continually monitored the RF power level measurements from the FITWD and updated its estimate of the local noise environment using a slew-rate limited update to the RF event threshold level. This slew-rate limited threshold adjustment allowed the trigger level to adapt to time varying system gain characteristics such as those that can occur in low noise amplifiers over temperature gradients. When a measured power level over the threshold (typically set at 2 dB over the current background estimate) is detected as being sustained for longer than a defined trigger time, such as 3 s, an RFI event was declared and raw samples are logged to the secure digital (SD) card of the Pi3 until either the termination of the event or the exhaustion of available disk space. The triggering threshold was allowed to vary during events to prevent flooding the available disk space with one long single event, preferring instead to attempt to gather multiple distinct events.

#### 2.1.7. Post-Mission Decoding and Result Production

A common challenge that arose when attempting to maximize the use of disk space for storing intermediate frequency (IF) data was that one was forced to adopt some proprietary or non-standard data format. Although this had the distinct advantage of achieving very high lossless compression (when compared to the use of native machine types), it could pose a challenge when attempting to share or distribute this data. In some data formats, including the one described here, the particular front-end configuration data was embedded with the IF data itself. This had the advantage of ensuring that the configuration of the data could not be lost or somehow dissociated with the data itself, and could enable dynamic reconfiguration; however, it added to the decoding complexity. Achieving widespread support for a bespoke data format was difficult as the bit-level manipulation of packed data was complicated and error-prone. Fortunately, this challenge was not unique to this front-end, and a concerted effort had been made by the community to find a solution.

Many commercial systems use some form of custom data format, including GNSS simulators, record and playback systems, and other software-defined-radio tools. To accommodate the ever-growing range of formats, an effort has been made by Institute of Navigation Software Defined Radio working group to develop a standardized way of describing such data. A “metadata” schema has been defined that is used to describe the packed binary format in a standardized way such that the description of can be conveyed between devices in an unambiguous manner. The standard uses extensible markup language (XML) to describe three different aspects of the data: the data collection scenario, the radio-frequency configuration of the data, and the bit-level packing of the data. At present, the standard has been presented for consideration by the Institute of Navigation and as part of this effort a normative reference implementation has been developed, which implements a decoder from packed binary data to native machine types. Interested readers can find this code at Reference [20]. To date, the standard has been applied to a wide range of commercial radio front-ends and a number of GNSS record-and-playback systems and constellation simulators.

## 3. Results

In this section we will discuss the deployment plan of the system, as well as example results that are representative of the field data collected so far in the project.

### 3.1. Deployment

The FITWD units were designed to be extremely simple to deploy, requiring only an antenna and power connection to operate. When RFI events were detected, an indicator light illuminated green, which subsequently turned red days, weeks, or months later depending on when the disk had filled to capacity. The SD cards were used for data storage and exchange, but given that monitors have been sent to multiple locations in Norway, multiple locations in the Netherlands, Germany, and soon France, future evolution of the system is intended to include internet connectivity and automated data recovery.

Typically, the units had been connected to antennas that are adjacent to busy local roads as most RFI sources were expected to be illegal personal privacy device (PPD)-type vehicle borne jammers, and despite the gain pattern of the antenna typically attenuating sources from below the antenna, they remained strong enough to be detected. In total, eight monitoring units have been produced and in the process of deployment through Europe and Scandinavia as of the time of writing this document.

In other cases, such as will be discussed in the next subsection, the units were deployed to a closed research campus at a European Space Agency (ESA) research facility, where passport checks and gated security were present, no busy roads were adjacent within kilometers, and no uninvited vehicles were permitted, yet jamming events were observed. Due to their radio-frequency signature (a chirp-like spectrum) similar to those reported in Reference [21] and the time of day that they occur, it is thought that these jamming signals were most likely originating from taxies or courier vehicles.

### 3.2. Example Results

**Figure 6:**
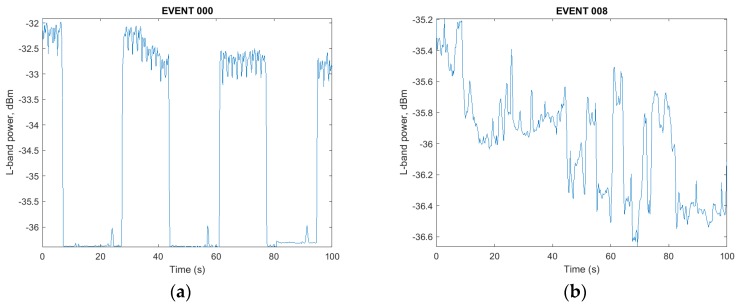
(**a**) Pulsed adjacent band transmitter; (**b**) In-band jammer preventing satellite acquisition.

Despite being deployed in an access-controlled research complex, located kilometers from the closest busy road, one FITWD monitor detected both in-band and adjacent-band interference. The adjacent band interference was thought to be due to one or more satellite uplinks located in the same complex that periodically transmitted at relatively high power levels in an azimuth that intersected the roof mounted GNSS antenna or had a reflection that did so. These types of events were characterized by rapid power level excursions as measured by the integrated RF power meter, but there was a complete absence of disturbance in the produced waterfall plots of the GNSS L1/E1 and L5/E5a spectra that were produced by post processing of the captured data. Per Figure 6a, the aggregate power level visible through the GNSS antennas pre-filters rose from below −36 dBm to −32 dBm, but due to the wide dynamic power range of the analog first stages not being violated and the sharp cutoff of the sixth order IF filters, no impact was observed in either the waterfall nor subsequent GNSS SDR processing to recover C/N0 levels.

In contrast, the event in Figure 6b was characterized by a sudden onset of a local RFI source that experienced power level variation as it moved through the local environment. A subsequent analysis step, which produces a waterfall plot, also showed changes to the local spectrum, which indicated the presence of a wideband interference source.

**Figure 7:**
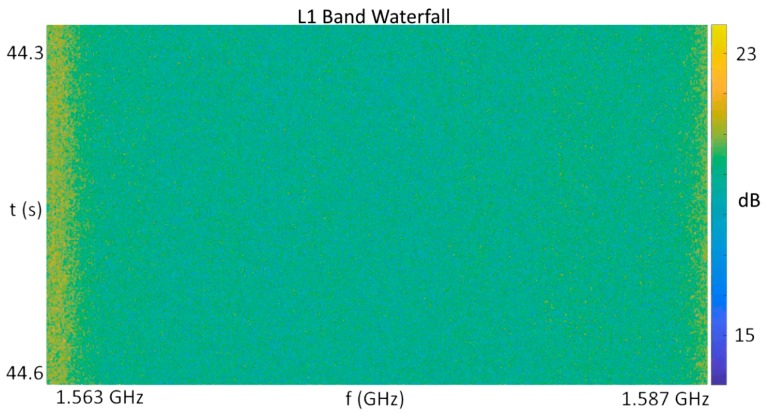
L1 band waterfall plot in nominal conditions.

A result of note in the case of these events was that the L5/E5a signal band remained free of any detectable jamming, indicating that the emitter blocking the reception of GNSS satellites targetted only the L1 band. When viewed over time in a “waterfall” style plot of frequency versus time versus intensity in Figure 7, the nominal conditions at this location resulted in slight elevation of the lower band edge over the midband, but a relatively consistent power density over both time and frequency otherwise.

**Figure 8:**
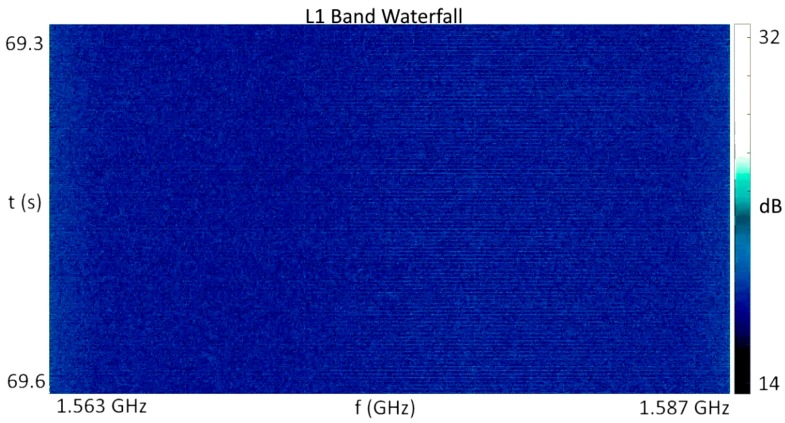
L1 band waterfall plot during wideband chirp interference, corresponding to Figure 6b.

**Figure 9:**
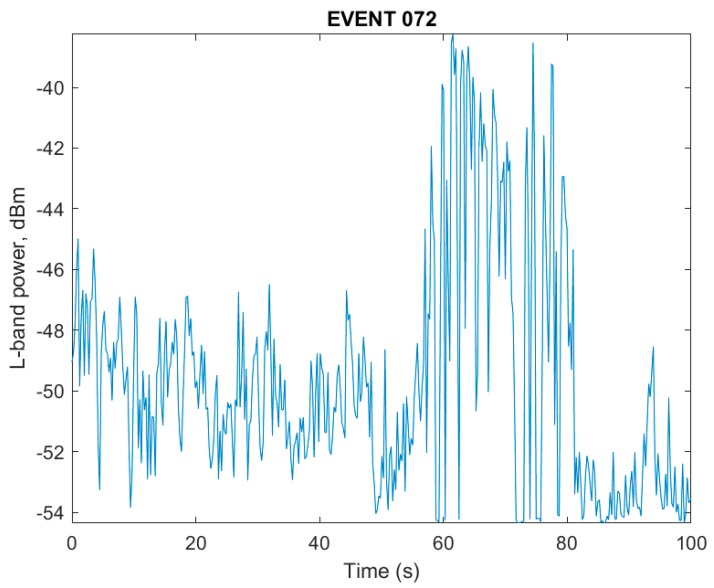
Pulsed L5 interference source.

**Figure 10:**
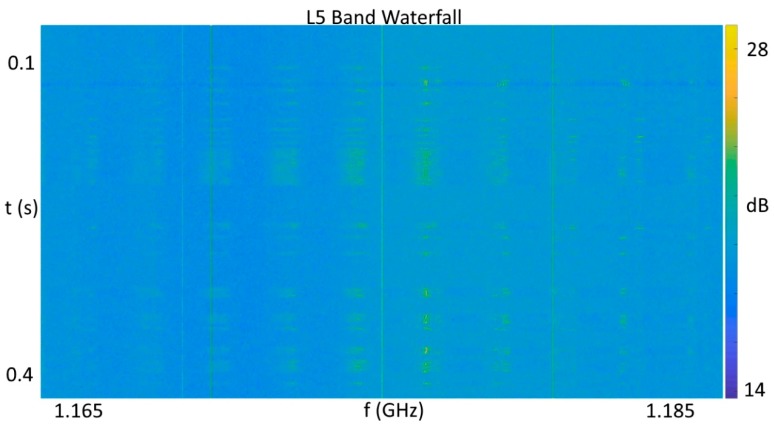
L5 band waterfall plot during pulsed interference, corresponding to Figure 9.

In contrast, the jamming event captured in Figure 8 shows multiple effects of wideband jamming present. First, the dynamic range of the rendered intensity levels increased by 10 dB as portions of the band were driven to saturation, while others are driven towards zero, and the normally raised power levels at the band edges were flattened out completely due to saturation of the single bit of dynamic range. The lateral striations in the spectrum in Figure 8 show the chirp transitioning across the band. Attempts to acquire L1 band GPS satellites during the pulsed adjacent band power event were successful, while attempts to acquire satellites during the depicted event from Figure 8 failed completely. During a separate RFI event impacting only the L5 band, for which the RFI power meter output is shown in Figure 9, the pulsed interference pattern shown in Figure 10 would intermittently saturate the single bit quantization as indicated by the dark band near the top of the Figure 10. It is not presently known if this emission was due to a legitimate, accidental, or malicious emission, but the concentration of signal power just above 1176.45, combined with the pulsed high power level, is an obvious concern.

Subsequent processing in a GNSS SDR written by one of the authors showed nominal tracking of L1 signals with C/N0 values up to 49 dB-Hz during the adjacent band pulsed power event spanning Figure 6a. In contrast, the same GNSS SDR was unable to acquire L1 signals during the event spanning Figure 6b.

## 4. Discussion

The deployed FITWD RFI monitors have detected several hundred events, with a small minority of these taking on the characteristics of an L-band jammer or PPD. In all but a few cases, no effects have been observed in the L5/E5a portion of the GNSS band, indicating that for the time being, the primary risk of RFI was carried by L1 receivers, and that having a second reception frequency may allow for fallback to the second frequency in most (though not all) cases. A secondary observation has been that even when using GNSS antennas that provide pre-filtering of out-of-band signals and a separate pre-filter to block the frequencies between L5 and L1 as well as those above L1, the system was still too sensitive to adjacent band transmissions.

While the design concepts behind the FITWD have led to a low-cost yet capable quad band front-end with implicit support for the synchronization of peripheral sensors and detection of L-band power excursions, at least two changes are desirable to improve the performance and reduce the false alarm rate of the system. To improve the performance, it is desirable to increase the quantization scheme from single-bit to at least four-bit, with the understanding that this will drive up system cost by at a minimum requiring a much more expensive single board computer and storage media to support the increased volume of data. To reduce false alarm rates, an additional stage of pre-filtering, preferably utilizing surface acoustic wave (SAW) or ceramic filters tailored to exactly the bands of interest for the RFI monitoring purposes that are called for.

## 5. Conclusions

This paper has reviewed the design and functionality of the FITWD front-end and how a subset of its features could be applied to produce a compact, low cost, low power, and highly portable RFI monitoring and logging system. It is shown that interference monitoring based purely on analogue-RF power monitoring is practical and cost-effective, and can be sufficiently sensitive to detect GNSS interference. It was illustrated that broadband power monitoring could be somewhat sensitive to out-of-band (wrt GNSS) interference. However, it was shown that this problem could be alleviated by subsequently processing IF samples gathered from each of the GNSS bands, allowing the monitor to discriminate between in-band and out-of-band interference. In line with its low-cost design, a one-bit digitization has been adopted, and has been shown to be effective, although it is evident that a multi-bit design and commensurately increased dynamic range would be desirable when analyzing RFI events.

While the full set of integrated sensors was not used in this effort, nor is the ability of the FITWD to phase coherently frequency hop between multiple L-band frequencies [11], the novel aspects of the synchronized and co-logged secondary sensors, along with the embedded configuration and status information, form a solid basis for myriad research activities.

## Figures and Tables

**Table 1:** FITWD operating parameter ranges and specific values used in presented data.

Parameter	Minimum	Utilized	Maximum
Channels	2	2	4
Mixer Frequency (MHz)	925	1176,1575	2175
Sampling rate (MHz)	2	24	54 ^1^
Two sided filter bandwidth (MHz)	8	23.66	80
Antenna power	Off	5 V @ 70 mA	24 V ^2^
Sampling Format and bits	1I,1Q	1I,1Q	1I,1Q
Secondary sensor payload capacity (kB/second)	3.5	42	93
Tuning resolution (MHz)	1	1	2 ^3^
Power Consumption (Watt)	1.8	2.0	2.2
Oscillator pulling range (ppm)	−5	+0.1	+5
Ceramic Filter pass-band (MHz)		1125–1675	
External filter band-stop (MHz) ^4^		1220–1525	
External filter lowpass (MHz) ^4^		0–1610	

^1^ In two-channel operating mode. ^2^ Protection from externally applied voltage. ^3^ Selectable, but undesirable. ^4^ External filters used in this work to limit triggering to GNSS L1 and L5 plus adjacent bands.
